# Muscle Imaging Approaches in Marinesco–Sjögren Syndrome: A Systematic Review and Two New Clinical Reports

**DOI:** 10.3390/children13030359

**Published:** 2026-03-02

**Authors:** Bianca Buchignani, Giada Vega, Rosa Pasquariello, Gemma Marinella, Michela Tosetti, Guja Astrea, Roberta Battini

**Affiliations:** 1 Department of Neuroscience, IRCCS Stella Maris Foundation, 56128 Pisa, Italy; bianca.buchignani@fsm.unipi.it (B.B.); rosa.pasquariello@fsm.unipi.it (R.P.); gemma.marinella@fsm.unipi.it (G.M.); roberta.battini@fsm.unipi.it (R.B.); 2 Department of Translational Research and of New Surgical and Medical Technologies, University of Pisa, 56126 Pisa, Italy; 3 Department of Clinical and Experimental Medicine, University of Pisa, 56126 Pisa, Italy; giada.vega@fsm.unipi.it; 4 Laboratory of Medical Physics and Magnetic Resonance, IRCCS Stella Maris Foundation, 56128 Pisa, Italy; michela.tosetti@fsm.unipi.it

**Keywords:** MSS myopathy, muscle imaging, MRI, SIL1-related myopathy

## Abstract

**Highlights:**

**What are the main findings?**

- Muscle imaging in Marinesco–Sjögren syndrome is largely unexplored
- Muscle imaging MRI can lead to the identification of early muscle involvement

**What are the implications of the main findings?**

- Muscle MRI should be implemented in the MSS diagnostic pathway to evaluate the rate of muscle involvement
- Muscle MRI can be used as a marker of disease progression

**Abstract:**

**Background**: Marinesco–Sjögren syndrome (MSS, MIM #248800) is a condition that is characterized by biallelic pathogenic variants in the *SIL1* gene. Manifestations include congenital cataracts, cerebellar ataxia, progressive muscle weakness and skeletal deformities, delay in psychomotor development, hypergonadotropic hypogonadism and short stature. Muscular involvement has been extensively discussed as a clinical finding but there is little literature on muscle imaging. The aim of this paper is to systematically review muscular imaging techniques in MSS reported in the literature, and to describe the clinical and imaging features of two pediatric subjects with MSS. **Methods**: Having searched through three electronic databases (PubMed, Scopus and Web of Science) two articles, written in English, describing twelve patients with MSS mutations on whom muscle MRI imaging was performed, were selected. In addition, two paediatric cases (brother and sister) with Marinesco–Sjögren syndrome (MSS) and MRI muscle findings were added. Data on type of study, cohort characteristics, type of mutation, neuromuscular signs and symptoms, imaging assessment, electrophysiological findings, biopsies, CNS symptoms, ocular signs and muscle imaging data were collected and stored in a table. **Results**: Of the 239 articles examined, only 3 used a muscle imaging technique to describe myopathy in MSS; one used a CT while another a muscle MRI. All 14 patients showed signs of fatty replacement. The infiltration mainly affected the lower limbs, but involvement in the upper limb was described in some adult patients. **Conclusions**: Performing a muscle MRI in MSS can lead to the early identification of muscle involvement and may be a useful biomarker to monitor disease progression.

## 1. Introduction

Marinesco–Sjögren syndrome (MSS, MIM #248800) is a condition characterized by biallelic pathogenic variants in the *SIL1* gene. This condition is primarily defined by a clinical tetrad: cerebellar ataxia, cataracts present from birth, impaired psychomotor development, and delay in psychomotor development. Motor involvement and cognitive delay are early signs of central nervous system involvement, but neuromuscular weakness and hypotrophy are also described as developing in the first decade. Other features include short stature, hypergonadotropic hypogonadism, bilateral cataracts, and skeletal deformities due to muscle weakness [1]. The progression of the disease involves a progressive decline in ambulation leading to the inability to walk independently, resulting in patients becoming wheelchair-bound [2], and surgical intervention for cataracts is often necessary [3]. As regards the laboratory exams routinely conducted, serum CK levels are normal or mildly elevated while the EMG reveals signs of dystrophic-myopathic involvement [4] and no peripheral neuropathy is reported in electron velocity conduction [5]. Muscle biopsy studies show muscle fiber necrosis and focal degeneration of both myofibrils and organelles, followed by an autophagic phenomenon [6,7]. Muscle biopsy findings under electron microscopy often show characteristic double-membrane formations and autophagic-type vacuoles. Membranous whorls associated with the nuclear periphery are also a common feature of the disease [8,9]. Diagnosis of MSS usually follows the observation of clinical signs and symptoms characterized by ataxic gait and early-onset bilateral cataracts, supported by brain magnetic resonance imaging (MRI) [10]. A small posterior fossa and marked cerebellar wasting—most notably involving the vermis—are standard neuroradiological signs of MSS. Furthermore, patients may exhibit supratentorial involvement, ranging from white matter lesions and cortical atrophy to structural anomalies of the pituitary gland [11]. Genetic analysis confirms the presence of the SIL1 mutation in the *SIL1* gene.

The progression of the disease can be explained by the function of SIL1, which is expressed in the endoplasmic reticulum, and whose function is to facilitate the release of ADP from the chaperone Binding immunoglobulin Protein (BiP), acting as a co-chaperone, thereby allowing BiP to release folded proteins. Thus, mutations that result in non-functional SIL1 lead to an accumulation of unfolded proteins. Moreover, both extracellular matrix remodeling and alteration have been reported in MSS, which therefore interfere with skeletal muscle, bone, and nervous system function [12].

There are many case reports reported in the literature regarding central nervous system involvement and clinical overview of the disease [13]. However, to date there is a lack of systematic reviews of muscle imaging and involvement. In a recent report [14], neuromuscular involvement is reported in over 90% of individuals with MSS, manifesting in early infancy with hypotonia during the first decade of life, with proximal and distal muscle weakness that, in turn, can lead to loss of ambulation.

The aim of this paper was to systematically review muscular imaging techniques in MSS reported in the literature, and to describe the clinical and muscle imaging features of two pediatric subjects with MSS.

## 2. Materials and Methods

### 2.1. Search Strategy and Selection Criteria

**Figure 1:**
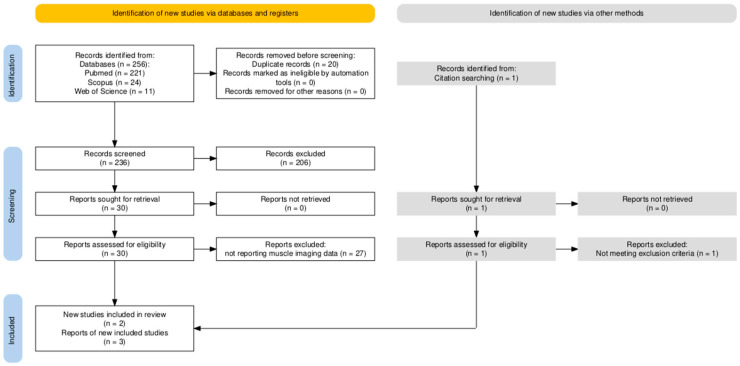
PRISMA flowchart of review.

A systematic search strategy was conducted following the Preferred Reporting Items for Systematic Reviews and Meta-Analyses (PRISMA) guidelines (Figure 1) [15].

Three electronic databases were used to carry out a search in the available literature: PubMed [16], Scopus [17], and Web of Science [18]. The search strategy, designed to include all fields (such as titles, abstracts, keywords), used strings adapted for each database: (marinesco sjogren syndrome [MeSH Terms]) AND (muscle imaging). Subsequently, the reference lists of the research papers and reviews that had been identified by the search were examined with the aim of finding additional studies that met our criteria. We used the research tool “Mendeley” [19] to collect all the results in a single library.

The selection of articles was based on the following inclusion criteria: (i) patients with MSS; (ii) description of the application of muscle imaging in patients. The exclusion criteria were: (i) duplicates; (ii) studies not concerning the aim of the paper; (iii) articles written in languages other than English, Italian and French; (iv) reports in the form of abstracts, reviews, theses, and conference papers.

Two researchers (G.V. and B.B.), working independently, compiled the full list of all the potentially eligible articles and their information. A third researcher (G.A) was called upon if any disagreement occurred.

The PRISMA flowchart shows the process of identification and selection of papers: 238 abstracts were initially obtained; one record was added by citation searching. Once duplicates were removed, 236 records were screened, 204 of which were excluded because they addressed different pathologies; 2 were excluded because they were written in languages other than English, Italian or French. Of the selected 30 articles, 27 were excluded because they did not report muscle imaging data. Ultimately, only three articles used muscle imaging to describe myopathy in MSS. Twelve patients were described in the selected studies. Other myopathic features were described in other articles, but no imaging method was applied (Figure 1).

### 2.2. Data Collection Process

Data were collected on the genetic, clinical, and muscle imaging features of the patients reported in each of the included studies. In particular, for each patient, we collected data on the type of study (retrospective/prospective), the cohort characteristics (n, F, M, mean age and sd), the type of mutation, myopathy signs and symptoms, brain imaging and muscle imaging (MRI/contrast/angioMRI/CT scan/angiography), type of involvement (%, site), location of muscle involvement, electrophysiological findings, biopsy and findings, CNS symptoms, imaging studies, ocular signs and symptoms, and other parameters that were assessed.

If data were not listed in the description, we wrote Not Applicable (NA). The review was not registered on PROSPERO. All data are included in Table 1.

### 2.3. Two Case Reports

We present two siblings of Pakistani origin, aged 10 years and 22 months, born from consanguineous parents. The elder sister was initially referred to the specialist when she was 5 years old for psychomotor delay and gait ataxia. Her developmental milestones were on time until 10 months of age, but she presented a delay in autonomous walking. When she was referred to the child neurologist she presented hypotonia, mild hypotrophy of her lower legs, hyporeflexia, and cerebellar signs such as ataxic gait, which was not yet autonomous, as well as tremor, dysmetria and nystagmus. She also presented with bilateral cataracts (removed with surgery) and hypostaturalism. Due to the clinical features, some blood tests were performed. CK was 293 U/L, and a brain MRI revealed cerebellar atrophy with both hemispheric and predominant vermin expression, characterized by cortico-subcortical hyperintensity in FLAIR sequences. In the intermediate-superior portions of the vermis and the cerebellar hemispheres, thinning of the superior and middle cerebellar peduncles and mild hypoplasia of the pons were also found.

An analysis for SIL1 was therefore performed, and a homozygous variant in SIL 1 was found: c.1276_1306del30 (p.Q426_L435del). She was diagnosed with MSS.

**Figure 2:**
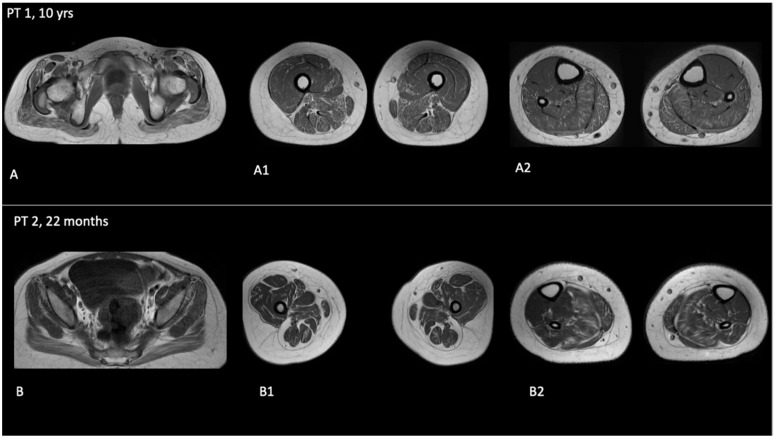
Muscle axial T1 weighted images from 3Tesla studies show PT1 and PT2 muscle involvement in the pelvis (**A**,**B**), thigh (**A1**,**B1**) and calf (**A2**,**B2**), respectively.

Following the diagnosis she underwent regular follow-ups and a surgical intervention for bilateral cataracts, and currently at the age of 10 she presents with generalized hypotonia, hyperlaxity, hyporeflexia, hypostaturalism, mild reduction of muscle trophism especially in the lower limbs, limb girdles and lower limb hyposthenia. She still presents an ataxic gait, but now she can walk independently without aids, dysmetria or action tremor. Two follow-up MRIs were repeated when she was six and ten years old, revealing brain stability. Due to the signs of neuromuscular involvement, at the age of ten, an MRI of the pelvis and lower limbs was also performed, which revealed mild-moderate fibro-adipose replacement involving gluteus maximus, gluteus medius, gluteus minimus, and tensor fasciae latae. The thigh imaging revealed involvement of the posterior compartment, particularly the adductor magnus and sartorius. As regards the leg compartment, diffused infiltration was found, except for the anterior and posterior tibialis. Hypotrophy of the gluteus maximus and tensor fasciae latae was also found (Figure 2).

Given the family history, the second sibling received the diagnosis of MSS during his fetal life. He presented with psychomotor delay, achieving head control by the age of 6 months. Currently, at the age of 19 months, he is still not able to sit independently, he presents axial hypotonia, hyperlaxity, and hyporeflexia, and antigravity movements are poor. Dysmetria, hypomimia and growth retardation are also present; no congenital cataract has yet been found.

Due to the severity of the clinical picture, a brain MRI was performed, which confirmed the suspected cerebellar atrophy with cortico-subcortical hyperintensity, similar to his sister’s neuroradiological picture. Moreover, a muscle MRI documented fibro-fatty degeneration with the same pattern as his sister (Figure 2).

## 3. Results

### Descriptive Findings

Two studies [20,21,22], reporting a total of 12 patients, were included in this systematic review. One article [21] reported a patient already described in a previous manuscript [22], so it was merged with the description of the clinical characteristics reported in Table 1. In the following analysis, we also include the two additional patients described in the present case report. Of the 14 patients included in this review, 9 had homozygosity over the 5q31 locus, which was found in Finland. Three had a homozygous frameshift insertion mutation, 936_937insG; p.(Leu313Alafs*39), while two presented a homozygosity deletion, c.1276_1306del30; p.(Gln426Serfs*22)

All the patients presented with psychomotor retardation and muscle weakness, and ataxic signs were found in all of them and bilateral cataracts in 11/14. Nine out of fourteen patients were able to walk at some stage of life; this information was not reported in two patients. The mean age of the patients was 36.2 ± 16 years old (range 19 months–51 years, median age 42.5 years); 8 patients were male and 6 were female. Mild to moderate intellectual disability was described in all patients, and hypergonadotropic hypogonadism was found in 10/12 of the adult patients. Bilateral cataracts were a common finding but articles did not specify the sample in which it was present.

The onset of neuromuscular symptoms was observed during the early stages of life in 11/14 patients; the age of symptom onset was not reported in 3 patients. Signs of neuromuscular involvement were found in neurological examination in all patients. All patients presented muscle weakness; 2/14 (14%) were not able to move, 4/14 (28%) presented severe weakness, while the remaining 8/14 (57%) presented mild weakness at different ages.

Seven patients underwent muscle biopsy at 18 months, 9, 12, 20, 30, 44 and 54 years, respectively, which showed myopathic-dystrophic findings with rimmed vacuoles in all cases. Myopathic changes were observed in the 10 adult patients who performed EMG.

As regards imaging, nine underwent a CT scan and five a muscle MRI; of the latter, only in three cases was a description of the pattern involvement or a complete analysis of imaging reported. In the imaging scans, the patients showed involvement both in the upper and lower limbs. As regards the lower limbs, early and severe fatty replacement was observed in the thigh in all muscles. However, this varied among the subjects. The following results focus on the findings of the ten patients where a description of full imaging data was reported [20,21], adding our two patients. Gluteus maximus, medius and tensor fasciae latae were described as being involved in all patients where the exam was performed. Additionally, in one of the pediatric patients, an involvement of the pelvic muscles was observed. Sartorius, gracilis and rectus femoris were reported with severe fatty replacement in all the adult patients, while they were still involved but less severely so in the pediatric population. Vastus intermedius and vastus medialis were less involved in 5/12 patients, but vastus lateralis was severely involved in all the patients. Semitendinosus was severely affected in 9/12 patients, while semimembranosus and biceps femoris showed less involvement in the adult population but were markedly affected in one pediatric patient. Adult patients showed an anterior–posterior involvement gradient while pediatric patients a posterior–anterior involvement gradient with sparing of the long adductor. In the leg, severe fatty replacement was observed in the peroneus, gastrocnemius and soleus in all patients, while the tibial anterior and extensor digitorum longus were less involved in most of the patients. In the upper limbs (nine patients studied with CT) the patterns were different due to the different degrees of involvement, from severe to no involvement. When the upper limb muscles were involved, the fatty replacement was mostly in the latissimus dorsi, serratus anterior and rhomboid muscles. Moreover, involvement of the paravertebral muscles was noted in 11/12 patients with different degrees of severity and with some correlation to the severity of scoliosis. See Table 1 for details.

## 4. Discussion

Marinesco–Sjögren Syndrome is a rare cause of autosomal recessive cerebellar ataxia that can present in the first stages of life, with psychomotor delay and congenital cataracts due to biallelic pathogenic variants in the *SIL1* gene. During the development of the disease, children and adults develop progressive muscle weakness, which may be severe and lead to an inability to walk without aids. The main imaging techniques to study this syndrome focus on brain imaging [13], even though severe cerebellar atrophy is not mandatory and a neuropathic phenotype with only slight changes in the posterior fossa was previously described by Reinhold et al. [23]. This review changes this point of view and focuses on another well-known entity of this syndrome, myopathy, and the limited number of contributions that focus on muscle imaging techniques to study muscle involvement. Although these are few in number, we state the possible value of muscle MRI in MSS and have added imaging studies of two previously unreported pediatric patients. Muscle MRI is a validated noninvasive technique that gradually replaced the use of CT scan due to the multiple radiation risks of the latter. Moreover, in the literature many studies analyzed muscle involvement using muscle biopsy in MSS, which is, however, an invasive technique and may not be key for follow-up.

Among the patients studied with an imaging technique, those previously reported in the literature were 12 adults [20,21], whereas the present article adds information from 2 paediatric patients. The described patients carried three different mutations; however, a clear genotype–phenotype correlation could not be found. Nonetheless, all the muscles were involved in most of the patients, but the gradient reported was somewhat different between the adult patients, involving homozygosity over the 5q31 locus as previously described in the literature, and the paediatric patients, carrying a homozygosity deletion c.1276_1306del30. The adults showed an anterior–posterior gradient in the thigh while the children presented a mild posterior–anterior gradient. It should be noted, however, that this gradient was only reported as qualitative data in 6/12 adult patients and no data were available in 2/12 adults, while it was most prominent in 1 of the 2 children. Moreover, as the muscle responsible for maintaining upright standing and helping antigravity movements is present in the posterior compartment of the thigh and leg, it is often the first affected in developmental delays. Thus, the posterior–anterior gradient found in the paediatric patients could at least partially explain the clinical picture of motor developmental delay onset.

Paravertebral and gluteal compartment muscles were severely affected both in the previously described adults and in the children we reported. These muscles, when affected, can lead to a Trendelenburg gait and posture, which was present in the girl described. Moreover, when the leg pattern of muscle involvement was described, the findings of the two pediatric patients were concordant with those reported in the adult patients. These showed the prevalence of involvement of the posterior compartment and relative muscle preservation of the anterior and posterior tibialis, suggesting a posterior–anterior fatty replacement gradient. Consistently with previous reported literature, the upper limbs, studied only in the adult population, showed less involvement than the lower limbs [20,21]. Even though in other types of congenital myopathies there is clear MRI pattern-recognition imaging that can also guide the diagnostic process, due to the low number of patients, a clear pattern in MSS cannot yet be identified and further studies with larger cohorts are needed to confirm these findings.

Muscle involvement in MSS has been previously described clinically, with lower limb atrophy and weakness identified through biopsy and imaging, showing a myopathic-dystrophic pattern [7,8], and by conducting preclinical studies [12,24]. These are devoted to explaining the pathogenic causes of this mechanism and can help researchers to develop innovative therapeutic targets. If in the past some authors had hypothesized and subsequently excluded that myopathic involvement could be at least partly secondary to hypogonadism [20], some recent findings suggest a mitochondrial energy deficit and a dis-adaptive cellular mechanism to manage oxidative stress in SIL1-deficient cells [24]. Currently, no therapy is available for MSS and a recent preclinical study showed no efficacy in the preclinical studies performed [25]. However, new therapeutic targets that focus on murine animal models are being analyzed [12], studying both neurodegeneration and muscle. Thus, natural history studies focusing on both neurodegeneration and muscle involvement have to be carried out so that a muscle MRI from early stages of the disease can help to identify muscle involvement in children who might be underestimated due to general developmental delay. More specifically, in these disorders where ataxia is often associated with hypotonia, the hypostenic component may be underestimated due to the ataxic features. Moreover, muscle involvement in MSS with an understanding of the strength deficit also has rehabilitative implications. Hence, promoting the importance of strengthening muscles as well as of improving balance may lead to earlier and more successful motor acquisition in these patients.

The use of muscle MRI as a follow-up measure is widely used in neuromuscular disorders [26]. However, it was only used in one of the studies in this review, where no variation in the muscle pattern after three years of treatment with testosterone and risedronate was observed, but an increase in bone density emerged [21]. This aspect is particularly relevant because symptoms of MSS can degenerate due to the accumulation of unfolded proteins, and may progress over time [24]. Assessment through follow-up muscle MRIs could help us to describe the degeneration of muscles and the progression of the disorder and to understand the different clinical severities. Moreover, it is known that changes on a muscle MRI often anticipate the onset of the clinical symptoms and this would help clinicians to strictly monitor some degenerative windows, which could possibly lead to early rehabilitative intervention [26]. Thus, the possibility of implementing muscle MRI in both the diagnosis and the follow-up of these disorders paves the way to a more systematic follow-up protocol. Furthermore, although the use of muscle imaging and MRI in the literature mostly relies on qualitative imaging, during follow-up new quantitative validated methods could also be useful to quantify the fat fraction involvement. Finally, the use of functional methods such as spectroscopy could improve knowledge of the myopathic process, not only by studying muscle involvement but also by investigating metabolic processes in MSS.

Some limitations should be addressed. The small number of studies included in this systematic review and the limited use of muscle imaging to assess muscle function in MSS shows the need for larger cohorts, which should include more patients and explore different techniques [20,21].

Having reported the two pediatric patients and reviewed the literature, we believe that muscle impairment can be present in the early stages of the disease in either mild or severe form [5,9], and that muscle MRI, being a non-invasive imaging technique, can help to record and monitor the progression of the pathology over time from an early stage of disease. Moreover, performing a muscle MRI could be useful in identifying the muscle involvement, which could be misdiagnosed by the presence of developmental delay, a non-specific symptom during the early stages of the disease, and could be masked by the presence of other neurological cerebellar signs. These could be considered the sole reason for an early clinical picture leading to mis-rehabilitation choices and misinterpretation of the evolutive trajectory.

If it is true that MRI plays an important role in neuromuscular disorders, performing a muscle MRI also in MSS can allow early identification of muscle involvement and may be useful for follow-up, thus avoiding more invasive tests such as biopsy. This data is all the more valuable since, in order to monitor the progression of cerebellar atrophy, these patients must undergo an MRI.

For all of these reasons, muscle MRI could be useful in the management of MSS.

## 5. Conclusions

MSS is a well-known clinical ataxia with neuromuscular involvement. However, there is a lack of standardized measures to follow-up the disease, and in preclinical models, increasing attention is given to muscle involvement. In this review we describe what is known about muscle imaging and we report the first two muscle MRI findings in two pediatric patients, suggesting the use of MRI as a possible biomarker for muscle involvement.

## Figures and Tables

**Table 1:** Summary of the laboratory and clinical features in the 14 patients with SIL1 and muscle imaging reports.

Authors	Cohort Characteristics (*n*; F/M; Mean Age)	Mutation	Weakness	Scoliosis	Amb.	MRI/ Contrast/ angioMRI/ CT Scan/ Angiography	CNV/EMG, Other Tests	Biopsy (Yes/No)	CNS Sympthoms	CNS Imaging	Ocular Signs and Sympthoms	Other Parameters Assessed
Mahjne et al., 2006 [20]	9; 4F/5M; 22–58 y	Homo over the 5q31 locus	Group 1: +; Group 2: ++; Group 3: +++	Group 1: −; Group 2: +; Group 3: ++	Group 1: Yes; Group 2 and 3: No	CT	EMG: myopathic features	Yes, 7 pts, myopathic-dystrophic with rimmed vacuoles in all cases	ID	Severe Dandy–Walker variant in the posterior fossae	Bilateral cataracts	Hypergonadotrophic hypogonadism, in some cases dysmorphism and skeletal abnormalities
Fujitake et al., 2011 [21]	3; 1F/2M; 38–48 y	Homo c. 936_937insG	+	NR	1 Yes 2 NR	MRI	1 EMG: myopathic change; 1 CNV: normal findings, reduced compound muscle action potential of the tibial nerves.	No	DD; mild ID, ataxia; mild cerebellar speech; 1 rotatory nystagmus	Marked cerebellar atrophy, especially the vermis. The pons was slightly atrophic and the fourth ventricle was enlarged	congenital cataract	1 primary hypogonadism, skeletal abnormalities, Low bone mass
Eriguchi et al., 2007 [22]												
This article	P1: 10 y, F; P2: 22 m, M	Homo c.1276_1306del30	P1: +; P2: +++	No	P1: Yes; P2: No	MRI	1 CNV normal (Patient 1)	No	P1: DD; mild ID; ataxia; P2: DD, dysmetria	P1: cerebellar atrophy: both hemispheric and vermin expression, characterized by cortico-subcortical hyperintensity. P2: cerebellar atrophy	P1: congenital cataract; P2: no	-

**Abbreviations and notes:** Homo, homozygous; F, female; M, male; m, months; y, years; Amb., Ambulant; −, not present, +, present; ID, Intellectual Disability; DD, Developmental delay; NR: Not reported. Muscle Weakness; +: present, ++: severe, +++: no movement. Scoliosis; −: absent, +: present, ++: severe.

## Data Availability

All procedures performed in this study were in accordance with the ethical standards of the institutional and/or national research committee and with the 1964 Helsinki declaration and its later amendments or comparable ethical standards.

## Abbreviations

The following abbreviations are used in this manuscript:

MSS	Marinesco–Sjögren syndrome
CNS	Central Nervous System
MRI	Magnetic Resonance Imaging
CT	Computed Tomography
CK	Creatine kinase
EMG	Electromyography
